# Die aktuelle Situation der kinder- und jugendpsychiatrischen Versorgung in Österreich im niedergelassenen Bereich

**DOI:** 10.1007/s40211-022-00437-w

**Published:** 2022-10-31

**Authors:** Doris Koubek, Helmut Krönke, Andreas Karwautz

**Affiliations:** 1 Kassenordination, für Kinder- und Jugendpsychiatrie, Linz, Österreich; 2 Praxis für Kinder- und Jugendpsychiatrie, Bundesfachgruppenobmann KJPP, Wien, Österreich; 3 Medizinische Universität Wien, Wien, Österreich

**Keywords:** Versorgung, Niederlassung, Ambulant, Kinder- und Jugendpsychiatrie, Österreich, Care, Outpatient care, Child and adoelscent psychiatry, Austria

## Abstract

Die kinder- und jugendpsychiatrische Versorgungslage in Österreich ist noch unvollständig und im Aufbau seit das Sonderfach gegründet wurde. Die Coronapandemie führt zu einem weiter erhöhten Versorgungsdruck auf die bestehenden Strukturen.

In dieser Arbeit soll der Status quo in der ambulanten Versorgung im niedergelassenen kassenärztlichen Bereich erhoben und zusammengefasst werden. Ein IST-Soll Vergleich soll die Defizite und Entwicklungsnotwendigkeiten aktuell dokumentieren.

Die 9 Bundesländer Österreichs sind bisher unterschiedlich weit in der Umsetzung einer Gesamtversorgung. Wir schließen mit Empfehlungen zur Umsetzung.

## Abstract (en)

The status of care in child and adoelscent psychiatry in Austria is still incomplete but increasing since the foundation of this medical specialty. Corona pandemia increases the pressure on the caring structures.

In this paper the current state of publically paid outpatient care is reviewed and summarized. Differences between current state and the desired/planned state of care will document existing deficits and needed developments.

The 9 federal states of Austria have made variable progress in developing the general child psychiatric care. We conclude with recommendations for further implementations.

In Österreich besteht ein System von Primär‑, Sekundär- und Tertiärversorgung, das historisch gewachsen ist und in den 70-er Jahren des letzten Jahrhunderts mit Tertiärversorgungsstrukturen begonnen hat. Diese arbeiteten sowohl universitär/forschend als auch stationär versorgend wie auch ambulant. Danach wurden in den meisten Bundesländern sekundäre Therapiezentren gegründet, die stationäre und ambulante Versorgung übernahmen. Inzwischen sind die sekundären Strukturen weiter im Aufbau, die sowohl im Rahmen von Ambulatorien als auch niedergelassenen Praxen die Patient:innen zu versorgen versuchen. Die Klienten werden dabei im Rahmen von kassenfinanzierten Ordinationen und Wahlarztordinationen in Einzelpraxen und vereinzelt auch Gruppenpraxen behandelt. Eine Primärversorgung fehlt bisher bzw. die Kassenarztordinationen und in geringem Umfang auch die Wahlarztordinationen übernehmen diese Aufgabe zusätzlich zu ihren Aufgaben in der Sekundärversorgung.

In den letzten 15 Jahren wurden gemäß dem politischen Willen und unter starker Beteiligung der Fachgesellschaft und der Bundes- und Landesvertreter:innen in den Ärztekammern in allen Bundesländern – mit Ausnahme des Burgenlandes – Verträge mit den Landesstellen der ÖGK abgeschlossen; zuletzt auch in der Steiermark. Auch bzw. besonders im niedergelassenen Bereich zeigt sich aber weiterhin die prekäre Situation der kinder- und jugendpsychiatrischen Versorgung in Österreich [1].

Ebenso sei hier erwähnt, dass es mit Einsetzen der Corona Pandemie zu deutlich mehr Anfragen und damit noch längeren Wartezeiten (durchschnittlich 3–6 Monate auf einen Ersttermin) in den Ordinationen gekommen ist. Derart lange Wartezeiten tragen zu einer Verschlechterung der Symptomatik aller Störungsbilder bei.

## Versorgungsbedarf vor und in der Corona-Pandemie

Zur Erhebung der Bedarfssituation vor Beginn der Corona-Pandemie sei hier die 2017 publizierte MHAT Studie [2] genannt, die diesbezüglich ein eindrückliches Bild zeichnet.

So lebten 2014 lt. Statistik Austria 780.185 Jugendliche im Alter zwischen 10 und 18 Jahren in Österreich. Unter Berücksichtigung der Behandlungsbedürftigkeit (14 % sicher akut behandlungsbedürftig (GAF < 70) und 8 % nicht akut behandlungsbedürftig (GAF > 70)) können wir auf der Basis der MHAT-Studie absolute Häufigkeiten von psychischen Erkrankungen bei Jugendlichen in Österreich (10–18 Jahre) für 2014 berechnen. So liegt bei etwa 171.000 Jugendlichen eine psychische Erkrankung vor, etwa 107.000 davon sind akut und sicher behandlungsbedürftig, die weiteren 64.000 sind behandlungsbedürftig aber nicht akut. Die häufigsten 5 Diagnosegruppen sind dabei Angststörungen, ADHS, Verhaltensstörungen, Anpassungsstörungen und Depressionen.

Unter Bedingungen der Corona-pandemie samt ihrem der Gesundheitssicherung dienenden Maßnahmen wie Lockdowns, Schulschließungen und Einschränkungen der Mobilität und des sozialen Zuganges erhöhten sich z. B. international die Vorstellungszahlen und die stationären Aufnahmen im Diagnosebereich der Essstörungen um 48 % [3]. Der rezente Kinder- und Jugendreport der DAK weist Steigerungsraten für die Neuerkrankungsraten von der präpandemischen Zeit 2018 bis 2021 für alle Störungen aus: 54 % für Essstörungen, 24 % für Angststörungen und 23 % für Depressionen [4].

Beide Studien zeigen während der Covid-Pandemie einen weiteren deutlich höheren Versorgungsdruck auf die kinder- und jugendpsychiatrischen Versorgungsstrukturen als in präpandemischen Zeiten.

## Versorgungsebenen

Der fachärztlich-niedergelassene Bereich ist im eigentlichen Sinne der Sekundärversorgung zuzuordnen. Dass dies im kinder- und jugendpsychiatrischen Bereich derzeit unmöglich ist, da in der Niederlassung zum Großteil alle 3 Versorgungsebenen (Prävention, Primärversorgung, Sekundärversorgung) abgedeckt werden (müssen), soll noch besprochen werden.

Die einzelnen gesetzlichen Verankerungen werden von den Sozialversicherungen und den Landesorganisationen der ÖÄK getragen. Der Gesamtvertrag der Kassenordinationen und beispielsweise demzufolge die Abrechnung einzelner Leistungen wird dementsprechend je nach Bundesland unterschiedlich geregelt. Es gibt Bemühungen darum, eine bundesweit einheitliche Linie zu finden.

„Kinder- und Jugendpsychiatrische Versorgung folgt dem Prinzip der Gemeindenähe und der Grundregel ambulant vor stationär. Es bedarf daher einer sorgfältigen, definierten und differenzierten Beschreibung dieses Segmentes der Versorgung“ [5, 6].

Aus Sicht der Niederlassung wurde dieser Prämisse bis dato kaum Beachtung geschenkt, da u. a. der Fachärzt:innenmangel in den Fachabteilungen, der durch eine „Pensionierungswelle“ in den nächsten 3–5 Jahren eklatant verschärft wird, immer im Vordergrund stand.

## Methode

### Bedarfserhebung

Wir erhoben den Bedarf an niedergelassenen Fachärzten und verglichen ihn mit dem IST-stand (1/2022). Die Kennzahl (ein niedergelassener Fachärzt*in pro 80.000 EW) entnehmen wir Fliedl et al. 2019 [5], den Anteil an Minderjährigen an der Bevölkerungsanzahl pro Bundesland der Statistik Austria [7].

## Ergebnisse

Wie in Tab. 1 ersichtlich ergibt sich bei 112 empfohlenen Kassenstellen ein Defizit von 74,25 fehlenden Kassenstellen bundesweit gemessen an einer Gesamtbevölkerungsanzahl von 9 Mio. EW (davon 1.730.794 Einwohner 0–19-jährige) in Ö (01.01.2022).

**Table 1:** 

Bundesland	IST Systemisierte Stellen	IST Besetzte Stellen	SOLL Systemisierte Stellen	SOLL minus IST	Offener Bedarf in %
Wien	9	9	24	15	62,5
NÖ	8	8	21	13	62
BGLD	0	0	4	4	100
OÖ	9	7	19	10	52,6
STMK	3	2	16	13	87,5
SLZBG	2	2	7	5	71,4
T	5	4	9	4	56
V	4,5	3,75	5	0,5	10
K	2	2	7	5	71,4
*Ö*	*40,5*	*37,75*	*112*	*74,25*	*66,29*

Aktuell sind derzeit 37,75 Kassenstellen besetzt, wobei es hierbei zu deutlichen Unterschieden in den Bundesländern kommt (Tab. 1).

Neben diesen konkreten Zahlen ist es wichtig darauf hinzuweisen, dass diese Bedarfsangaben immer auch in Relation zur erweiterten kinder- und jugendpsychiatrischen Versorgung in den entsprechenden Bundesländern zu sehen sind.

Hier sollen zur Illustration der aktuellen Situation zwei Beispiele herausgenommen werden.

Erstens: Im Bundesland Oberösterreich (288.195 0–18-Jährige Statistik Austria 01/2022 [7]) wird derzeit die kinder- und jugendpsychiatrische Versorgung inklusive Akutversorgung im vollstationären Bereich (54 Betten) in den beiden Standorten in Linz von insgesamt 8,2 Fachärzt:innen aufrechterhalten. Eine neu etablierte KJP in Grieskirchen mit 15 Betten trägt aufgrund fehlender Möglichkeit einer psychiatrischen Unterbringung nur beschränkt zur Akutversorgung schwer kranker Patient*innen bei. Ebenso wurden in OÖ diverse Jugendberatungsstellen geschaffen bzw. ein Ambulatorium ohne kinder- und jugendpsychiatrische Fachärzt:in bzw. in nicht nennenswertem Ausmaß (1 h fachärztliche Tätigkeit pro Woche im Ambulatorium). Man kann sich also vorstellen, in welchem Ausmaß unversorgte, zu einem nicht unbeträchtlichen Teil psychisch schwer kranke Kinder und Jugendliche in die Ordinationen drängen.

Zweitens: Im Bundesland Wien (350.695 0–18-jährige, Statistik Austria 01/2022, [7]) gibt es derzeit im vollstationären Bereich 13 Fachärzt:innen (1 Karenzstelle) an der UK (36 vollstationäre Betten) – diese sind jedoch nicht zur Gänze der Versorgung zuzuordnen, da die Universitätsklinik dem wissenschaftlichen Auftrag Folge leisten muss – und 3 (sic!) Fachärzt:innen an der Fachabteilung Klinik Hietzing – Standort Rosenhügel. 2 kinder- und jugendpsychiatrische Ambulatorien mit angeschlossener Tagesklinik tragen zwar maßgeblich zur Versorgung bei, trotzdem ist es eindeutig nachvollziehbar, dass der Versorgungsdruck auf die niedergelassenen Kolleg:innen enorm ist.

An dieser Stelle muss gesagt werden, dass die rezent iniziierte politische Debatte, Wahlarztordinationen abzuschaffen, in der kinder- und jugendpsychiatrischen Versorgungslandschaft obsolet ist, da diese Ordinationen derzeit besonders im Ballungsraum Wien einen erheblichen Beitrag an der Versorgung haben und Ihre Abschaffung in eine komplette Versorgungslücke hinterlassen würde.

## Aktuelle Situationsbeschreibung

Aktuell zeigt sich im niedergelassenen Bereich folgende Situation:

Nahezu alle Kolleg*innen mit Kassenstellen nennen hohe Patient*innenzahlen in den Ordinationen – pro niedergelassener Kassenärzt:in werden im Schnitt 850 Patient*innen p. a. versorgt, die in unterschiedlicher Frequenz die Ordination konsultieren. Pro Woche aufgeschlüsselt bedeutet dies für die meisten, dass es zu ca. 50 persönlichen Konsultationen kommt, hinzu kommen in etwa 50–70 weitere indirekte Kontakte wie Telefonate mit Institutionen – vor allem Schulen und Jugendhilfeeinrichtungen – sowie der Schriftverkehr (Befunderstellungen, Mailkontakte, etc.).

Diese Arbeit wird mit administrativer Unterstützung der Ordinationsassistent:innen von den Kolleg:innen in den Ordinationen allein getragen. Das heißt im konkreten Fall Diagnostik, Therapie(-planung), Helferkonferenzen, Familiengespräche u. ä. werden nicht im interdisziplinären Setting – wie in allen anderen Bereichen der kinder- und jugendpsychiatrischen Versorgung – durchgeführt, sondern werden alleine von den niedergelassenen Kolleg:innen bewältigt.

Ist etwa eine klinisch psychologische oder ergotherapeutische Diagnostik notwendig, muss an diese Berufsgruppen im niedergelassenen bzw. extramuralen Bereich zugewiesen werden, was zu weiteren Wartezeiten führt.

Am dringendsten fehlt es in der Niederlassung dabei an Sozialarbeiter:innen und Sozialpädagog:innen. Bei Familien aus sozioökonomisch schlechteren Verhältnissen ist somit die komplette sozialarbeiterische Tätigkeit und Vernetzungsarbeit allein durch die Fachärzt:in zu leisten – auch die chronisch überlasteten Mitarbeiter:innen der Jugendhilfe unterstützt nur in den dringendsten Fällen.

Besonders betont werden muss, dass der Versorgungsbereich der Niederlassung sich in einer Situation befindet, in der einerseits die Primärversorgungsebene zumeist fehlt, andererseits bzw. vor allem aufgrund der defizitären Versorgungssituation in den Fachabteilungen (Fachärzt:innenmangel, monatelange Aufenthaltsdauern aufgrund der schweren Krankheitsbilder, etc.), schwer kranke und stationspflichtige Patient:innen in den Kassenordinationen versorgt werden müssen!

Nahezu alle Kolleg:innen bundesweit berichten davon, dass es sehr schwierig, in manchen Bundesländern sogar unmöglich ist, Patient:innen in einer stationären Einrichtung zur Behandlung zuzuweisen. Hierbei seien vor allem Patient:innen erwähnt, die an Anorexia nervosa leiden. Eine leitliniengerechte Behandlung ist oft aufgrund fehlender Ressourcen vor Ort (prompte, kassenfinanzierte Diätologie und störungsspezifische Psychotherapie, engmaschige Kontrollen) unmöglich, eine Aggravierung der Symptomatik ist bei fehlenden stationären Behandlungsplätzen oftmals die Folge. Wenig bis gar nicht unterstützend erwiesen sich dabei diverse Einrichtungen (beratend, punktuell psychotherapeutisch), die keinerlei fachärztliche Behandlungsmöglichkeit anbieten, da sie ohne fachärztliche Leitung tätig sind.

Ebenso konsultieren Patient:innen die Kassenordinationen, die eine Behandlung in einem fachärztlich geführten Ambulatorium oder in einer kinder- und jugendpsychiatrischen Tagesklinik benötigen würden, hier seien beispielsweise Patient*innen mit monatelangem schulverweigerndem Verhalten erwähnt.

Auch der rehabilitative Bereich (Es existieren in Ö 96 belegbare Betten – 113 sind im Vollausbau geplant) für („Mental Health Rehabilitation“) bietet für die Niederlassung keine Entlastung, da einerseits das Behandlungsspektrum aufgrund der dort zumeist fehlenden Fachärzt:innen sehr eng gesetzt wird, andererseits diese Einrichtungen disloziert sind und somit dem Versorgungsanspruch nicht gerecht werden können.

Ein großes Problem sehen wir – neben der Versorgung der jugendpsychiatrischen Patient:innen – in der Versorgung der kinderpsychiatrischen Patient:innen. Hierbei gibt es bundesländerbezogen deutliche Unterschiede bezüglich der Angebote anderer ambulanter Einrichtungen (Ambulatorien, therapeutische Einrichtungen, etc.).

Wir wissen jedoch, dass Kinder, die mit multiplen Risikofaktoren aufwachsen (alleinerziehender Elternteil, sozioökonomische Benachteiligung, schlechte Wohnverhältnisse, etc.), ein deutlich höheres Risiko haben, als Erwachsene nicht nur psychisch zu erkranken, sondern auch vermehrt an chronischen körperlichen Erkrankungen leiden, wie z. B. Diabetes mellitus Ty II, KHK, etc. [8]. In diesem klassischen Primärversorgungsbereich sehen wir deutliche Defizite, die in den fachärztlichen KJP-Ordinationen nur rudimentär kompensiert werden können.

Spürbar wird in den Kassenordination zudem das Defizit an kassenfinanzierten Psychotherapieplätzen, zum Teil müssen Patient:innen bis zu über einem Jahr auf einen Platz warten, hierbei wurde jedoch noch keine störungsspezifische Psychotherapie mitbedacht. Diese sehr ungünstige Situation kann von den Kolleg:innen in den Ordinationen in der Regel nicht ausgeglichen werden.

## Diskussion

Zusammenfassend ist hervorzuheben, dass es aus der Sicht der Niederlassung eine Erneuerung bzw. eine Reform eines Gesamtkonzeptes für die kinder- und jugendpsychiatrische Versorgung braucht.

Am Beispiel der prekären Situation in den Ordinationen sieht man deutlich, dass nicht allein die Anzahl der benötigten Kassenfacharztstellen ausschlaggebend ist, sondern vor allem auch die Versorgungssituation in den Fachabteilungen und in anderen ambulanten Strukturen bzw. natürlich auch das Fehlen von spezialisierten, fachärztlich geleiteten Einrichtungen! Auch bzw. besonders im niedergelassenen Bereich zeigt sich auch weiterhin die prekäre Situation der kinder- und jugendpsychiatrischen Versorgung in Österreich. Und dies nicht nur in absoluten Zahlen – wir sprechen hier von 71,5 fehlenden Kassenstellen bundesweit – sondern auch relativ gesehen, da der Mangel an Fachärzt:innen in den Fachabteilung und das Fehlen einer klassischen Primärversorgung (im Sinne präventivmedizinischer Einrichtungen) naturgemäß eine deutliche Mehrbelastung für die niedergelassenen Fachärzt:innen bedeutet.

Die Corona-Pandemie führte zu einer Zunahme von Prävalenz und Inanspruchnahme von Behandlungsplätzen, was die Versorgungssituation weiter unter Druck brachte und bringt und die multidisziplinäre Kooperation sehr erschwert.

## Umsetzungsempfehlungen

In der Praxis der Niederlassung braucht es dringend die Unterstützung von *Sozialarbeiter:innen und Sozialpädagog:innen*. Ebenso benötigt wird ein direkter Zugriff auf kassenfinanzierte *Psychotherapieplätze*, in weiterer Folge gilt dies ebenso für andere *funktionelle Therapien* und für die *Diätologie*.

Darüber hinaus wird derzeit leider das Potenzial, weitere Kinder- und Jugendpsychiater:innen in einer *Lehrpraxis* für den dafür vorgesehenen Zeitraum (1 Jahr) auszubilden, nicht genützt, da die niedergelassenen Kolleg:innen die Kosten allein zu tragen hätten und schlicht die notwendige Zeit für die Supervision der jungen Kolleg:innen aufgrund der hohen Patient:innenzahlen fehlt. Das ist insofern bedauerlich, da in kaum einer anderen Versorgungsstruktur so viele Patient:innen gesehen werden, die das gesamte Störungsbild- und Behandlungsspektrum sowie das gesamte Altersspektrum abdecken. Zudem müssen Primärstrukturen und Präventionsangebote ausgebaut werden, Frühintervention und digitale Angebote hätten hier zunehmenden Stellenwert [9]. Auch die Integration der Kinder- und Jugendpsychiatrie in Primärversorgungszentren (wie z. B. von Puspök et al. [10] gefordert) bleibt ein Desiderat.

Die kinder- und jugendpsychiatrische Versorgung hat bisher die im Strukturplan Gesundheit für Österreich geforderten Versorgungsziele nicht erreicht. Ein dringender Blick politischer Entscheidungsträger auf diesen wichtigen Bereich der Versorgung der Kinder und Jugendlichen mit psychischen Störungen verbunden mit dem politischen Willen und der politischen Potenz für eine Umsetzung der Behebung der gegebenen Defizite ist essentiell. Unsere Kollegenschaft ist bereit, ihren Teil beizutragen.
